# PRL-3 and E-cadherin show mutual interactions and participate in lymph node metastasis formation in gastric cancer

**DOI:** 10.1007/s13277-014-1855-7

**Published:** 2014-04-03

**Authors:** Anna Pryczynicz, Katarzyna Guzińska-Ustymowicz, Katarzyna Niewiarowska, Dariusz Cepowicz, Andrzej Kemona

**Affiliations:** 1 Department of General Pathomorphology, Medical University of Bialystok, ul. Waszyngtona 13, 15-269 Bialystok, Poland; 2 Second Department of General and Gastroenterological Surgery, Medical University of Bialystok, Bialystok, Poland

**Keywords:** E-cadherin, Gastric cancer, Metastases, PRL-3

## Abstract

E-cadherin, a transmembrane adhesion molecule, and phosphatase of regenerating liver 3 (PRL-3) protein, a member of the family of tyrosine phosphatases, seem to be responsible for cancer cell migration. Therefore, the study objective was to determine a correlation between PRL-3 and E-cadherin, to assess their expression in neoplastic tissue and normal mucosa of the stomach, to analyze their effect on cancer advancement, and to evaluate their potential as prognostic markers in gastric cancer. The expressions of PRL-3 and E-cadherin were assessed immunohistochemically in 71 patients with gastric cancer. Positive expression of PRL-3 was observed in 42.2 % of gastric cancer cases, whereas E-cadherin expression was abnormal in 38 % of cases. The study revealed that the positive PRL-3 expression and abnormal E-cadherin expression were associated with mucinous gastric carcinoma and lymph node involvement. The former was also related to the infiltrating type of tumor and abnormal E-cadherin expression. The expression of PRL-3, but not of E-cadherin, was associated with shorter survival of patients. PRL-3 and E-cadherin exhibit interactions in gastric cancer and are involved in the formation of lymph node metastases. The PRL-3 protein can be an independent predictive factor of overall survival in gastric cancer patients.

## Introduction

The phosphatase of regenerating liver 3 (PRL-3) protein belongs to the family of tyrosine phosphatases, with a unique COOH-terminal prenylation motif, and it is thus involved in a major reaction for the cell, i.e., dephosphorylation of tyrosine residues deactivating enzymes. Although its physiological role is poorly investigated, literature data suggest that PRL-3 takes part in neoformation, i.e., in migration, metastasizing, and angiogenesis [1, 2]. However, factors that regulate PRL-3 expression as well as its enzymes are not well known, and researchers are still searching for pathways and processes associated with the protein involvement. A few studies have revealed a link between PRL-3 and proteins responsible for cytoskeleton rebuilding [3–7]. Regulation of cell adhesion is another mechanism of the protein in the promotion of cancer cell invasion and metastasizing [8, 9]. The PRL-3 is also involved in tumor growth through the mechanism of epithelial-mesenchymal transformation (EMT). PRL-3 activates the Akt pathway, which results in glycogen synthase kinase 3β (GSK-3β) inactivation and then in overexpression of mesenchymal markers—vimentin, fibronectin, and Snail—and a decrease in γ-catenins, integrin β3, and E-cadherin responsible for cell adhesion [10].

E-cadherin is a transmembrane protein, which, in normal epithelium, is responsible for intracellular interactions. Its cytoplasmic domain interacts with β-catenin or γ-catenin. The complex that is formed binds α-catenin, which directly affects the cytoskeleton [11, 12]. In cancers, dysfunction of E-cadherin induced by lack of the cytoplasmic domain (no interaction with catenins) or extracellular domain (no interaction with adjacent cells with a simultaneous accumulation of catenins) and changes in mutations are observed. These disorders can lead to the detachment of cancer cells from the primary tumor mass and thus increase their invasiveness [13, 14].

Considering the similar role of both proteins in the migration of cancer cells and EMT, the objective of the current study was to determine the correlation between the PRL-3 protein and E-cadherin, to assess their expression in cancer tissue and in normal gastric mucosa, as well as to investigate their effect on tumor stage. Also, the prognostic potential of both proteins was assessed in gastric cancer.

## Material and methods

The study involved 71 patients with gastric cancer treated surgically in the Second Department of General Surgery and Gastroenterology in the years 2005–2010. As a control, healthy gastric mucosa was collected from a stomach fragment removed during therapeutic surgery. The postoperative material was fixed in buffered and paraffin-embedded formalin. From paraffin blocks, 4-μm sections were cut off and stained with hematoxylin-eosin (H + E). Routine histopathological analysis included determination of tumor histological type, malignancy grade (G), anatomoclinical stage (pT), and lymph node metastases. Gastric cancers were also divided according to Lauren’s classification [15], Goseki’s classification [16], and Borrmann’s classification [17]. Also, the presence of *Helicobacter pylori* infection was assessed in Giemsa-stained preparations.

### Immunohistochemical analysis

Tissue blocks were cut using a microtome into 4-μm-thick sections on silanized glasses. The sections were deparaffinized in xylenes and hydrated in alcohols. In order to exhibit antigen, the tissue sections were heated in a microwave for 15 min in citrate buffer (pH = 6.0). Then, they were incubated with 0.5 % hydrogen peroxide in methanol to block endogenous peroxidase and, next, with mouse anti-PRL-3 antibody (clone 3B6; Attogen Biomedical Research, USA; 1:500 dilution) overnight at 4 °C and with mouse monoclonal anti-E-cadherin antibody (clone 36B5). Following streptavidin-biotin reaction (biotinylated secondary antibody, streptavidin-HRP; Novocastra, UK), the antigen-antibody complex was visualized by application of chromogen 3,3′-diaminobenzidine (DAB; Novocastra, UK). The expressions of the proteins were assessed using the semiquantitative method, which defined PRL-3 expression as positive when the reaction was visible in more than 5 % of cancer cells and negative when there was no reaction or it was present in <5 % of cells. For E-cadherin, the reaction was normal when the membranous expression of the protein was present in >30 % of cells; it was abnormal when negative or when membranous expression was present in <30 % of cells or in the case of cytoplasmic expression. Positive reaction was calculated in at least 500 cancer cells in each tissue section using a light microscope (×400).

The statistical analysis was based on Fisher’s test and *χ* ^2^ test. Log-rank test which is according to Kaplan-Meier survival analysis approach was employed to compare the overall survival rate of patients. A Cox proportional hazard model was used for univariate and multivariate analyses. A *p* value of <0.05 was considered statistically significant. The statistics was performed using STATISTICA 10 (Poland).

## Results

### PRL-3 and E-cadherin expression in normal gastric mucosa and in gastric cancer

**Fig. 1:**
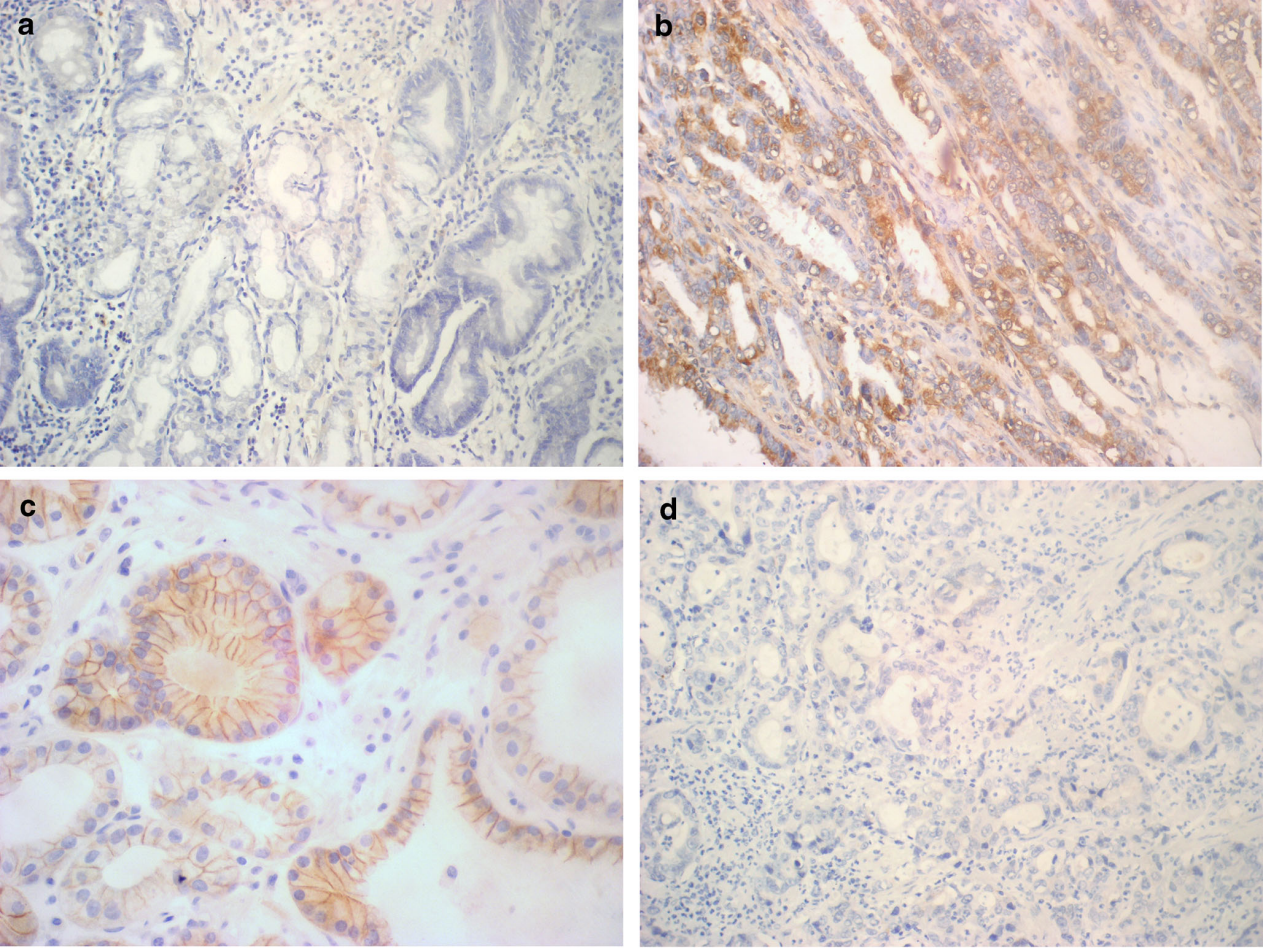
Immunohistochemical staining. **a** PRL-3 expression in the gastric mucosa. **b** Positive cytoplasmic PRL-3 reaction in gastric cancer cells. **c** Cytoplasmic membrane expression of E-cadherin in the gastric mucosa. **d** Negative of E-cadherin expression in gastric cancer cells. Original magnification: ×200 and ×400

Normal gastric mucosa did not show PRL-3 expression, whereas E-cadherin was present on the cytoplasmic membrane of most glandular cells. The neoplastic tissue had a positive expression of PRL-3 in 42.2 % (30/71) of gastric cancer cases, whereas abnormal expression of E-cadherin was observed in 38 % (27/71) of cases (Fig. 1).

### PRL-3 and E-cadherin expression in correlation to clinicopathological parameters in gastric cancer

Statistical analysis revealed no correlations of the expression of PRL-3 and E-cadherin with sex, age, tumor location, infiltration depth (pT), malignancy grade, tumor type according to Goseki and Lauren, and the presence of *H. pylori* infection. The positive expression of PRL-3 and abnormal expression of E-cadherin were shown to be associated with a mucinous type of gastric cancer (*p* = 0.002 and *p* = 0.012, respectively). Positive expression of PRL-3 was also related with the infiltrating type of tumor in Borrmann’s classification (*p* = 0.037). Patients with local lymph node involvement also had increased PRL-3 and abnormal E-cadherin expression in primary tumor (*p* < 0.001). Moreover, positive PRL expression was associated with abnormal expression of E-cadherin (*p* = 0.007) (Table 1).

**Table 1:** Relationship between the expressions of PRL-3 and E-cadherin proteins and clinicopathological parameters in gastric cancer

Variables	PRL-3 expression		*p* value	E-cadherin expression		*p* value
	Absent	Present		Abnormal	Normal	
Age						
≤ 50	10	10	0.415	12	12	0.358
> 50	31	20		15	32	
Gender						
Male	28	21	0.880	21	30	0.591
Female	13	9		6	14	
Location						
Upper 1/3	2	4	0.114	1	1	0.568
Middle 1/3	16	14		14	16	
Lower 1/3	23	12		13	26	
Depth of invasion						
T1	5	1	0.114	1	5	0.794
T2	11	6		6	8	
T3	25	23		21	30	
Histological differentiation						
Moderately differentiated	20	9	0.115	12	23	0.585
Poorly differentiated	21	21		15	21	
Hp						
Adenocarcinoma	36	17	*0.002*	21	42	*0.012*
Adenocarcinoma mucinosum	5	13		10	5	
Goseki’s classification						
I	7	4	0.254	3	5	0.188
II	8	10		9	11	
III	5	5		6	5	
IV	14	18		17	13	
Lauren’s classification						
Intestinal type	31	16	0.051	16	35	0.086
Diffuse type	10	14		11	9	
Borrmann’s classification						
I (polypoid)	4	1	*0.037*	3	4	0.935
II (fungating)	8	4		8	13	
III (ulcerated)	26	17		19	27	
IV (infiltrative)	3	8		7	10	
Lymph node metastasis						
Absent	40	9	*<0.001*	10	45	*<0.001*
Present	1	21		17	1	
*Helicobacter pylori* infection						
Absent	16	18	0.373	15	20	0.320
Present	20	17		12	24	
E-cadherin expression						
Abnormal	9	14	*0.007*	–	–	–
Normal	28	10				

Significant relationship is marked in italics

Missing data were removed in pairs

### Correlation of PRL-3 and E-cadherin expression with patients’ survival

**Fig. 2:**
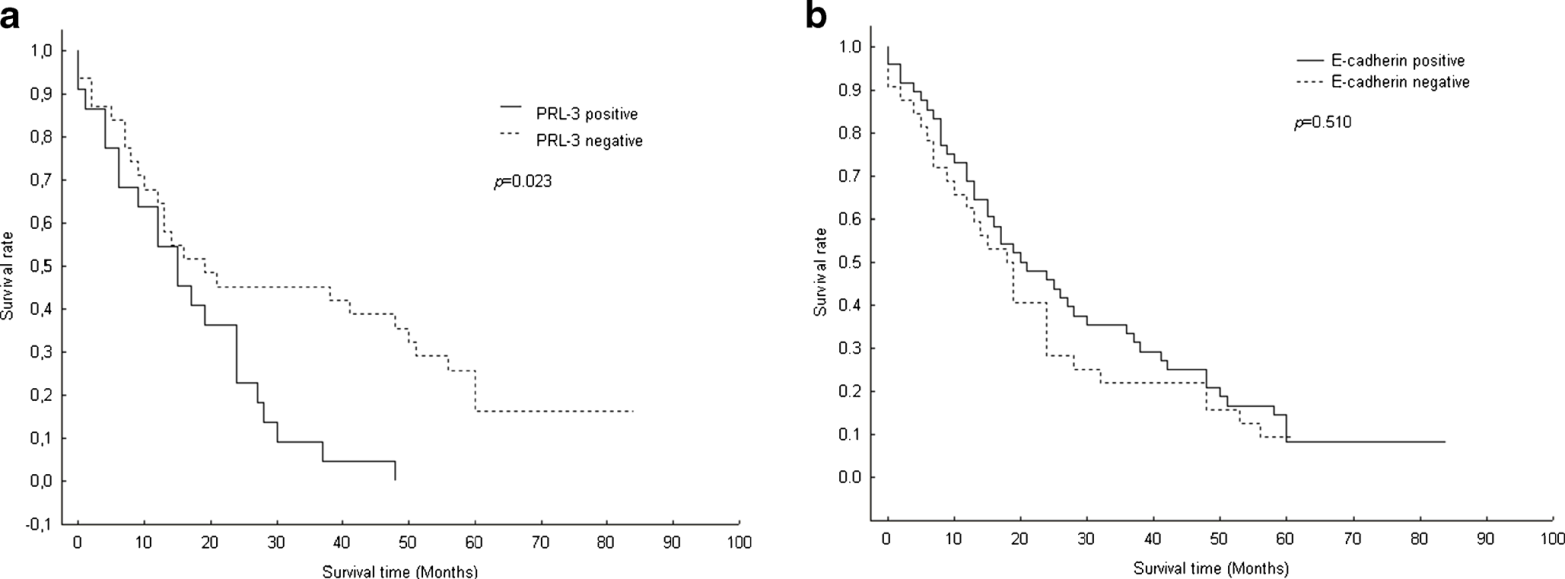
Postoperative overall survival of patients with gastric cancer. Comparison of postoperative survival according to the expressions of PRL-3 and E-cadherin

Overall assessment of patients’ survival showed a lack of correlation between the expression of E-cadherin and survival rate (*p* = 0.510). However, the postoperative time of overall survival in patients with positive PRL-3 expression was markedly shorter (*p* = 0.023) (Fig. 2).

### Prognostic factors of gastric cancer

As shown by univariate Cox regression analysis, tumor location, histological malignancy grade, tumor type in Lauren’s classification, and positive PRL-3 expression were essential predictive factors of overall survival among gastric cancer patients (*p* = 0.046, 0.004, 0.021, and 0.002, respectively). Malignancy grade, tumor type in Lauren’s classification, and positive PRL-3 expression were independent predictive factors of overall survival for these patients (*p* = 0.006, 0.011, and 0.001, respectively) (Table 2).

**Table 2:** Univariate and multivariate analysis of clinicopathological factors in gastric cancer

Variables	Univariate *p* value	Multivariate *p* value	Hazard ratio	95 % CI
Age (≤50 vs. >50)	0.081	–	1.600	0.942–2.715
Gender (male vs. female)	0.641	–	1.137	0.660–1.959
Location (upper 1/3, middle 1/3, vs. lower 1/3)	0.873	–	1.042	0.627–1.730
Depth of invasion (T1, T2, vs. T3)	*0.046*	0.078	1.852	1.008–3.403
Histological differentiation (moderately diff. vs. poorly diff.)	0.641	–	1.145	0.646–2.029
Hp (adc. vs. adc. mucinosum)	*0.004*	*0.006*	0.293	0.127–0.678
Goseki’s classification (I and III vs. II and IV)	0.864	–	1.073	0.476–2.419
Lauren’s classification (intestinal type vs. diffuse type)	*0.021*	*0.011*	2.422	1.140–5.143
Borrmann’s classification (I and II vs. III and IV)	0.068	–	0.587	0.331–1.040
Lymph node metastasis (absent vs. present)	0.372	–	0.705	0.327–1.519
*Helicobacter pylori* infection (absent vs. present)	0.713	–	1.109	0.635–1.937
PRL-3 expression (absent vs. present)	*0.002*	*0.001*	4.059	1.615–10.198
E-cadherin expression (abnormal vs. normal)	0.318	–	0.713	0.367–1.384

Significant relationship is marked in italics

Borrmann’s classification: I (polypoid), II (fungating), III (ulcerated), and IV (infiltrative)

*diff.* differentiated, *adc.* adenocarcinoma, *CI* confidence interval

## Discussion

EMT is a physiological process of growing embryos during which epithelial cells are transformed into mesenchymal cells. In a similar way, cancer cells of epithelial origin change into mobile mesenchymal cells. Cell junctions are lost, and cancer cells in this form are able to migrate to remote parts of the body. After being released from the primary tumor, they invade the surrounding tissues, penetrate lymphatic or blood vessels, and migrate through the vascular wall, where they eventually settle, proliferate, and induce angiogenesis. There, they become transformed into epithelial cells and adhere tightly to one another to form a metastatic tumor [18]. The ability of cancer cells to disseminate from the primary tumor to lymph nodes and to the nearest and distant tissues and organs is a major feature of malignant neoplasms and the main cause of therapeutic failure. Since tumor stage pTNM is the key factor, with the greatest significance in the treatment of gastric cancer patients, research into markers involved in metastasizing has been intensified. E-cadherin, a transmembrane adhesion molecule and PRL-3 belonging to the family of tyrosine phosphatases seem to be particularly responsible for the migration of cancer cells. Wang et al. [19] have been the first to suggest the involvement of PRL-3 in EMT. They have put forward the hypothesis that PRL-3 activates the Akt pathway through direct inhibition of PTEN (inhibitor for PI3K), which results in GSK-3β inactivation. Next, Liu et al. [20] have presented evidence for PRL-3 involvement in EMT via cadherin-related signaling pathway. Most likely, PRL-3 plays a major role in direct inhibition of the expression of E-cadherin and CDH22 [20]. In our study, we analyzed the immunohistochemical correlation between the expression of PRL-3 and E-cadherin in gastric cancer and observed a correlation between increased PRL-3 expression and abnormal E-cadherin expression (*p* = 0.007), which indicates that they may interact.

We also compared the relationship of PRL-3 and E-cadherin with clinicopathological parameters in gastric cancer. We revealed a correlation of positive PRL-3 and abnormal E-cadherin with mucinous type of gastric cancer. Similar observations have been reported by other authors, indicating a relationship between these proteins and signet ring cell-type carcinoma [21, 22]. It is likely that an abnormal expression of E-cadherin and an overexpression of PRL-3 are associated with the loss of cell junctions and loosening of cells in this histological type. Importantly, both proteins were found to correlate with the presence of local lymph node metastases. Thus, PRL-3 and E-cadherin seem to exert an extremely significant effect on the spread of gastric cancer through the lymphatic pathway. Our findings are compatible with earlier literature data [21–24].

We also analyzed whether the proteins could be prognostic factors in gastric cancer. Only the positive expression of PRL-3 was found to correlate with shorter survival of patients. We also demonstrated that PRL-3 was an independent prognostic factor and could be used as a therapeutic target in this cancer. Ooki et al. [24] have additionally observed that PRL-3 overexpression is an independent prognostic factor in patients with gastric cancer without metastases to local lymph nodes as compared to patients with lymph node involvement. Although other studies, including the research conducted by Li et al. [25], have shown that also an abnormal expression of E-cadherin can be a strong independent prognostic factor of the overall survival of patients with gastric cancer, we did not observe such correlations for the expression of E-cadherin.

In conclusion, our study found a significant correlation between PRL-3 protein and E-cadherin. However, it is the PRL-3 protein which seems to be more important in the growth of gastric cancer.
